# TLR4-Targeting Therapeutics: Structural Basis and Computer-Aided Drug Discovery Approaches

**DOI:** 10.3390/molecules25030627

**Published:** 2020-01-31

**Authors:** Qurat ul Ain, Maria Batool, Sangdun Choi

**Affiliations:** Department of Molecular Science and Technology, Ajou University, Suwon 16499, Korea; ainne.w@gmail.com (Q.u.A.); mariabatool.28@gmail.com (M.B.)

**Keywords:** TLR4, computer-aided drug discovery, agonist, antagonist, virtual screening, molecular dynamics

## Abstract

The integration of computational techniques into drug development has led to a substantial increase in the knowledge of structural, chemical, and biological data. These techniques are useful for handling the big data generated by empirical and clinical studies. Over the last few years, computer-aided drug discovery methods such as virtual screening, pharmacophore modeling, quantitative structure-activity relationship analysis, and molecular docking have been employed by pharmaceutical companies and academic researchers for the development of pharmacologically active drugs. Toll-like receptors (TLRs) play a vital role in various inflammatory, autoimmune, and neurodegenerative disorders such as sepsis, rheumatoid arthritis, inflammatory bowel disease, Alzheimer’s disease, multiple sclerosis, cancer, and systemic lupus erythematosus. TLRs, particularly TLR4, have been identified as potential drug targets for the treatment of these diseases, and several relevant compounds are under preclinical and clinical evaluation. This review covers the reported computational studies and techniques that have provided insights into TLR4-targeting therapeutics. Furthermore, this article provides an overview of the computational methods that can benefit a broad audience in this field and help with the development of novel drugs for TLR-related disorders.

## 1. Introduction

**Figure 1:**
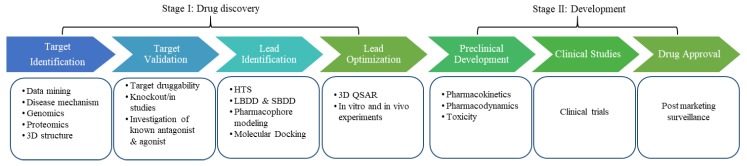
The drug discovery and development pipeline. The initial step in drug discovery is target identification; the aim is to find a potential target via different approaches like genomics and proteomics. The next step is target validation, which classifies the molecular target and evaluates whether it is suitable for drug development by various in vivo and in vitro methods. Various techniques such as high-throughput screening (HTS), ligand-based drug design (LBDD), pharmacophore modeling, molecular docking, and structure-based drug design (SBDD) are employed to identify hit compounds. These hit molecules are then evaluated and modified to improve the activity of the lead compound (e.g., by quantitative SAR [QSAR] analysis). Prior to human clinical trials, preclinical studies are carried out that provide detailed knowledge on the toxicity and appropriate dose of drugs. Based on these findings, it is decided whether the drug is ready to be tested in humans. Clinical trials are the final stage of the drug development process. Once clinical trials are over, the successful drug is approved by the FDA and becomes available on the market for clinical use.

Toll-like receptor 4 (TLR4) belongs to the pattern recognition receptor family, which plays a key role in the human defense mechanism and responds to invading pathogens with high selectivity and sensitivity [1,2]. TLR4 is sensitive to pathogen-associated molecular patterns (PAMPs) such as lipopolysaccharide (LPS) and lipo-oligosaccharide. Moreover, TLR4 recognizes PAMPs from fungi, viruses, and mycoplasmas [3]. In addition to PAMPs, TLR4 can be activated by certain endogenous ligands produced due to tissue injury and/or inflammation [3,4]. This receptor–ligand interaction initiates an intracellular signaling cascade that leads to the subsequent proinflammatory response [5]. Thus, due to the involvement of TLR4 in various pathological conditions, it is considered a potential therapeutic target for drug development. Many drugs targeting TLR4 are already in clinical trials, and the two Food and Drug Administration (FDA)-approved drugs named Bacillus Calmette–Guerin (a vaccine used against tuberculosis) and monophosphoryl lipid A (MPLA; used for the treatment of bladder and cervical cancer) are available on the market [6]. Nonetheless, there is a need for new drugs for life-threating diseases involving TLR4 dysregulation. Thus, the hunt for a potential drug targeting TLR4 is still in progress. Over the past decades, computer-aided drug discovery (CADD) has made tremendous progress in the development of drugs including small organic molecules, aptamers, peptides, and antibodies. CADD is a cost-effective method that has accelerated the drug development process by reducing the total timespan from target identification to hit discovery and optimization [7]. Generally, CADD is categorized into two main approaches: structure-based drug design (SBDD) [8] and ligand-based drug design (LBDD) [9,10,11]. In SBDD, the structural information on the target molecule is used to identify hotspot residues and key interactions that are crucial for their biological activity [7]. Such information can be helpful for designing new drug candidates to modulate the function of proteins involved in a disease. In the absence of protein three-dimensional (3D) structure, the LBDD method is employed, which takes into account the information on known ligands for the specific target. Structure-activity relationship (SAR) analysis is one of the applications of LBDD method. In SAR analysis, the chemical structure and biological activity of known ligands can be utilized to design new drugs or optimize known drugs to increase their efficacy [10]. The main steps in the drug discovery pipeline are target identification and validation followed by lead discovery and optimization (Figure 1). In next stage, the optimized hit is tested empirically, whereby toxicity and pharmacokinetic properties are evaluated. Afterwards, nontoxic and effective hits proceed to preclinical and clinical trials, and the most effective drug that passes the clinical trials is distributed in the market [7]. This review focuses on the activation mechanism and structural details of TLR4 and most importantly, the therapeutic importance of TLR4 along with the progress in drug development through computational techniques. Furthermore, light is shed on various computational studies that have addressed the importance of TLR4 by means of various state-of-the-art computational tools.

## 2. Structure and Interaction Interfaces of TLR4

TLR4 is a transmembrane receptor that belongs to the superfamily of leucine-rich repeat (LRR)-containing proteins. LRRs are 20–29 residues long sequence motifs present in various proteins and provide unique structural features for protein–protein interaction. The full-length structure of TLR4 is composed of three domains: an ectodomain, transmembrane helical domain, and intracellular Toll-interleukin-1 receptor (TIR) domain. Thus far, none of full-length crystal structures of TLR4 has been resolved, but the structures of individual domains have been reported. Crystal structure of the extracellular domain (ECD) of human TLR4 in complex with myeloid differentiation factor 2 (MD2) and LPS has been resolved [12]. The ECD consists of 608 amino acid (aa) residues and has a horseshoe-like shape comprising a concave and convex surface [13]. Parallel β-sheets collectively make up the concave surface of the ECD, while the convex surface consists of loops and 3_10_ helices [14]. The ECD is responsible for the recognition of specific structural patterns in invading microbial molecules, where the binding of these molecules with the receptor leads to the activation and dimerization of TLR4 [5,15].

**Figure 2:**
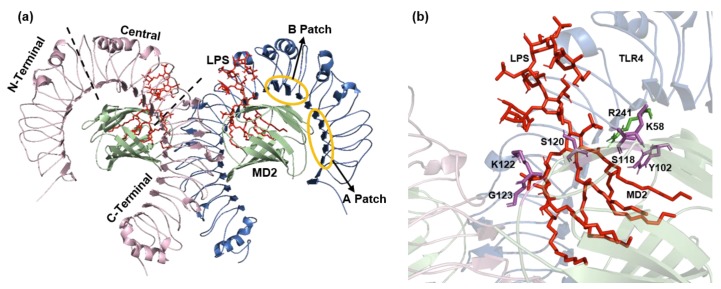
TLR4-MD2-LPS complex (PDB ID: 3FXI). (**a**) The ECD is divided into three subdomains: N-terminal, central, and C-terminal domains. The evolutionary conserved patches A and B resides in the N-terminal domain and central domain, respectively. (**b**) Key residues involved in the binding of LPS with TLR4-MD2 complex. TLR4 residue R241 is shown in green color and MD2 residues K58, Y102, S118, S120, K122, and G123 shown in magenta color. LPS is represented by red color.

The ECD of human TLR4 (27–631 aa) is further classified into three subdomains: N-terminal domain, central domain, and C-terminal domain (Figure 2a). The N-terminal domain is a highly hydrophobic region that extends from residue 27 to residue 201 and contains LRR modules 1 through 6. The central domain contains a hypervariable region and is important for LPS recognition. It is composed of LRR modules 7 to 12. Lastly, the C-terminal domain contains LRR modules 13 to 22 [12,13]. β-Sheets of the C-terminal domain are 28% larger in radii as compared to the N-terminal domain. The N-terminal and central domains of TLR4 collectively constitute a primary contact surface comprising two evolutionarily and chemically distant regions, the A patch and B patch. These patches enable charge complementarity to its coreceptor MD2 for binding, forming a stable heterodimer [12,16]. The A patch is evolutionarily conserved and negatively charged and interacts with a positively charged MD2 surface. By contrast, patch B is positively charged and interacts with the negatively charged surface of MD2. Any mutation in the primary interface can disrupt TLR4–MD2 binding [13]. The transmembrane domain of human TLR4 (632–652 aa) forms a helical structure of 21 residues followed by loop and a juxtamembrane helix. The intracellular TIR domain is composed of 187 aa and is responsible for the initiation of the downstream signaling cascade [5,17].

MD2 has a β-cup fold structure composed of two antiparallel β-sheets separated from each other and forming a hydrophobic pocket, which is ideal for the binding of ligands such as LPS. The β-cup fold structure of MD2 is different from the immunoglobulin fold because it lacks a conserved disulfide bridge that connects the two β-sheets thus forming a large internal pocket. The internal surface of the pocket is made up of hydrophobic residues, and the outer region contains the positively charged residues that promote the LPS binding (Figure 2b) [13,18]. Electrostatic interactions, hydrogen bonds, and a few hydrophobic residues contribute to the binding of MD2 to TLR4.

## 3. The Signaling Mechanism of TLR4 and Its Role in Inflammatory Diseases

**Figure 3:**
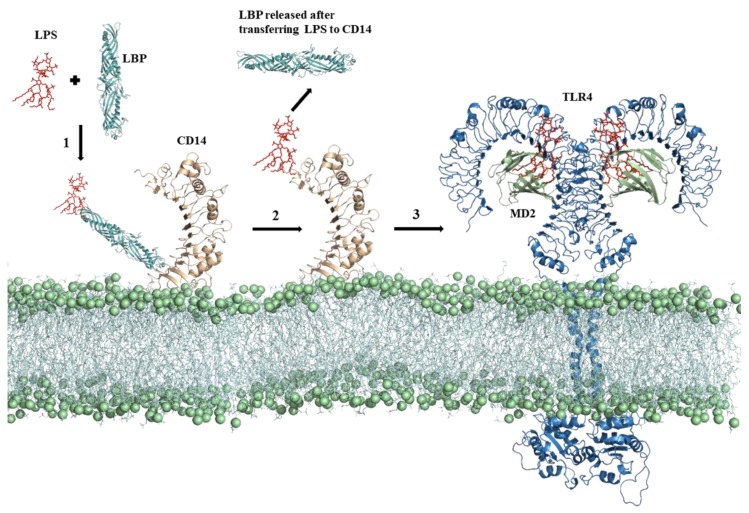
The binding mechanism of LPS for TLR4 activation. A series of events takes place prior to LPS recognition by the TLR4-MD2 complex. (1) LBP extracts LPS from the bacterial membrane and (2) transfers it to membrane anchored CD14, where it binds to the hydrophobic pocket located at the N terminus and forms a monomeric complex. (3) CD14 facilitates LPS transfer to the TLR4-MD2 complex where it initiated the intracellular pathway.

**Figure 4:**
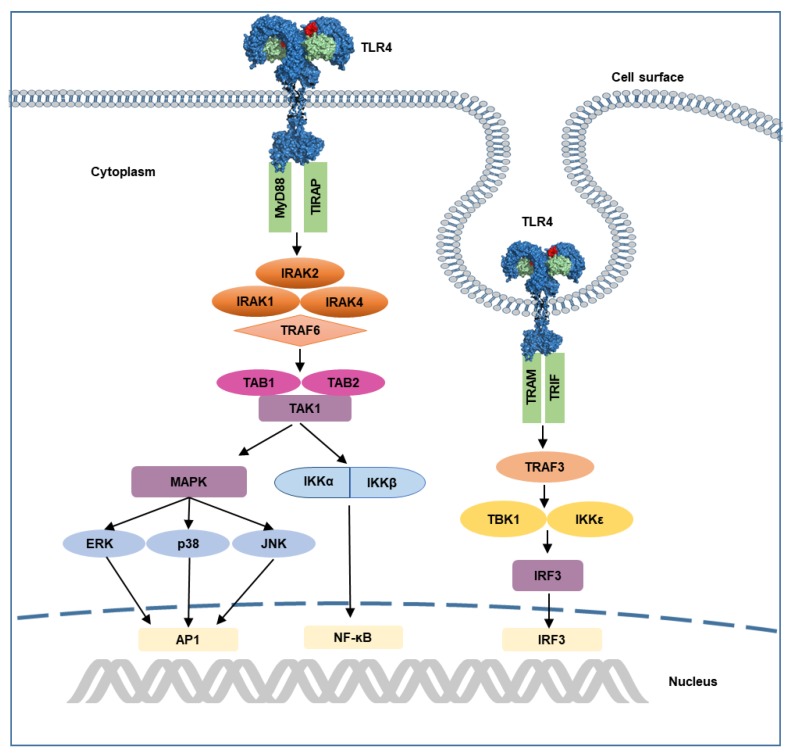
Illustration of TLR4 signaling pathway. Recognition of LPS activates MyD88-dependent and TRIF-dependent pathways. In MyD88-dependent pathway, recruitment of MyD88 by TIRAP initiates the interaction of IRAKs and TRAF6, that leads to the activation of transcription factors. Endocytosis of TLR4 initiates TRIF-dependent signaling, which involves recruitment of TRIF and TRAM, that leads to subsequent induction of TBK1 and IKKε, as well as activation of transcription factor IRF3. AP1, activated protein 1; ERK, extracellular-regulated kinase, IRAK, interleukin receptor-associated kinase; IKK, inhibitor of ĸ B kinase; IRF3, interferon response factor 3; JNK, c-Jun N-terminal kinase; MAPK, mitogen-activated protein kinase; MyD88, myeloid differentiation primary response protein 88; NF-ĸB, nuclear factor ĸB; p38, protein 38; TAK1, transforming growth factor β-activated kinase 1; TBK1, TANK-binding kinase 1; TIRAM, TRIF-related adaptor molecule; TIRAP, TIR domain-containing adaptor protein; TRAF, tumor necrosis factor receptor-associated factor.

A release of LPS from invading bacteria is an early sign of infection and is capable of inducing a strong immunological response [16]. LPS is composed of three structural components: (I) a conserved hydrophobic lipid A component, (II) a branched hydrophilic core polysaccharide chain, and (III) a hydrophilic repeating oligosaccharide side chain [15]. The engagement of TLR4 by LPS has a complicated mechanism and involves several proteins including LPS-binding protein (LBP), cluster of differentiation 14 (CD14), and MD2. LBP extracts LPS from the bacterial membrane and then transfers it to CD14, which assists with the transfer of LPS to the TLR4–MD2 complex [15,19,20]. Upon LPS recognition, the TLR4–MD2 homo-heterodimer forms that triggers the downstream signaling cascade. This process is schematically presented in Figure 3. TLR4 has a distinctive capability of initiating two signaling cascades: the myeloid differentiation primary response protein 88 (MyD88)-dependent pathway and TIR domain-containing adaptor inducing interferon-β (TRIF)-dependent (MyD88-independent) pathway (Figure 4) [15]. In the former, TLR4-MD2 dimerization at the cell surface recruits adaptor proteins TIR domain-containing adaptor protein (TIRAP) and MyD88. MyD88 interacts with the IL-1R–associated kinase (IRAK) complex, which recruits tumor necrosis factor receptor–associated factor 6 (TRAF-6) [21,22,23]. It promotes the activation of transcription of nuclear factor-κB (NF-ĸB) genes and proinflammatory cytokines tumor necrosis factor-α (TNF-α) and interleukin-6 (IL-6) [24,25,26]. Endocytosis of TLR4-MD2 complex activates the MyD88-independent pathway in the endosomal compartment [27]. Instead of TIRAP, TRIF-related adaptor molecule (TRAM) recruits TRIF and switches TRAF3 on. TRAF3 is responsible for downstream induction of TANK-binding kinase 1 (TBK1) and IKKε leading to the activation of transcription factor IRF3 and type I interferon [28,29]. The MyD88-independent pathway stimulates the late-phase induction of a mitogen-activated protein kinase (MAPK) and NF-ĸB [30].

Generally, inflammation has a protective role in the affected area; however, an unnecessary host response to various exogenous or endogenous molecules can lead to serious health problems [31]. Clinical data shows that any dysregulation of TLR4-mediated signaling triggered by PAMPs and damage-associated molecular patterns (DAMPs) may cause autoimmune, cardiovascular, neurological, and infectious diseases [32,33,34,35,36]. TLR4 also recognizes DAMPs including high-mobility group protein 1 (HMGB1), heparin sulfate, hyaluronic acid, heat shock proteins, and oxidized phospholipids [3,4].

In addition to immune cells, tumor cells express TLR4, where it acts as a double-edged sword on tumorigenesis. Various lines of evidence have revealed that TLR4 engagement within a tumor microenvironment can promote either antitumor immunity or tumor progression [37,38,39,40,41]. It has been reported that TNF-α boosts intratumoral penetration of chemotherapeutic agents and acts as an adjuvant for chemotherapy. Moreover, as discussed elsewhere, activation of two distinct signaling pathways by TLR4 and TNF-α secretion makes TLR4 a promising target for cancer immunotherapy [42,43]. Nevertheless, a better understanding of the mechanism of action of TLR4 in cancer and in other diseases is necessary to develop potent and successful drug candidates.

## 4. The Role of Computational Techniques in TLR4-based Therapeutics

Computational techniques have been extensively used in drug development to find potent therapeutic compounds [44]. So far, no effective compound against TLR4-mediated diseases is available on the market. The computational strategies can help with the discovery of novel compounds with promising therapeutic effects. Various computational methods employed in the drug discovery process include virtual screening (VS), quantitative SAR analysis, pharmacophore-based screening, molecular docking, and molecular dynamics (MD) simulations.

### 4.1. Target Identification and Structure Determination

The initial step in the first stage of the drug development process is target identification [7]. To identify the druggable biological molecule responsible for specific disease is an important step followed by functional characterization of the target molecule [45]. The characteristics of a druggable target molecule are safe (non-toxic), efficacious, and meet clinical requirements. Various computational methods like panel docking [46], or data mining/machine learning [47] and experimental techniques such as expression profiling [48], cell-based assays, and functional screening, are available for identification and selection of potent target molecules. Target validation involves confirmation of role of target molecule in the disease and whether its activity can be modulated using a small molecule drug or biologic [49]. Once the potential target molecule is identified, next step is structure determination. 3D structure information of therapeutically important targets is crucial for the development of a potent drug. Many integrated structural biology techniques are available such as cryo-electron microscopy, nuclear magnetic resonance method, and X-ray crystallography but in the absence of solution structure, computational techniques are available. Computational methods include homology modeling, threading, and ab initio modeling. Homology modeling is more reliable approach among in silico methods as it considers the structural information of homologous proteins. The predicted structures are further validated using various validation tools such as Ramachandran plot [7].

As described above, TLR4 is a promising therapeutic target because of its involvement in the pathogenesis of various diseases. To date, 12 solution structures of human TLR4 are available in the Protein Data Bank (PDB) and are listed in Table 1. Most of the crystal structures are those of the ECD N terminus, only one crystal structure of the C terminus of the ectodomain is available, and a few structures of the full-length ECD have been reported too. Furthermore, two NMR structures of the transmembrane helix are resolved, but unfortunately full-length structure of TLR4 is not determined yet.

A study has been conducted to identify the interaction surface of the TLR4-TIR domain with the TIR domain of adaptor proteins MAL and TRAM as well as the TIR-TIR interaction for TLR4 dimerization [52]. Because a solution structure of the TIR domain is not available, a homology model was constructed computationally. The homology model was energy minimized and optimized on Web servers for further use [52]. Three putative binding interfaces (for MAL, TRAM, and TLR4 TIR) were identified by alanine scanning mutagenesis. These sites were confirmed via protein-protein docking. The two sites include residues of the BB loop, whereas a third binding site is present on the paradoxical surface. Furthermore, these results were confirmed by in vitro assays [52].

To predict the full-length structure of TLR4, including the ectodomain, transmembrane, and cytoplasmic domains, a homology modeling technique has been used [53]. Online modeling server SWISS-MODEL was utilized to construct a full-length model of the TLR4 protein. All three domains were modeled independently and were combined into the single model by establishing peptide bonds between the residues present at N and C termini of flanking domains. For this purpose, Discovery studio Visualizer was used. Next, these peptide bonds were optimized, and the full-length model was energy minimized in the Gromacs software [53].

### 4.2. Lead Identification and Optimization

Lead identification is the next step in stage I of the drug discovery process. For identification of novel hits, VS is a robust approach [54]. In VS, a database of millions of compounds is screened and docked into a target molecule to choose hits with better binding affinity. There are two types of VS, i.e., ligand-based VS and structure-based VS. Ligand-based VS includes SAR analysis, QSAR analysis, and pharmacophore-based screening of huge libraries of active compounds. The highly active scaffolds from these libraries are identified considering various factors, such as descriptors, a similarity index, and a consensus pharmacophore [55]. By contrast, in structure-based VS, the 3D structure of a target protein and its active binding site are analyzed. The druglike molecules are docked into the binding site of the protein via suitable docking algorithms. Then, on the basis of scoring functions, the binding affinity of the docked complexes is evaluated [7]. The top hits are next optimized by structural modifications. While the main scaffold is preserved, side chains can be replaced to increase stability and efficacy. This task can be accomplished by computational methods and synthesized experimentally. The top hits are then tested in vitro and in vivo for confirmation of the results.

#### 4.2.1. Small-Molecule Inhibitors of TLR4: Lead Identification & Preclinical Studies

Two small-molecule compounds, C11 and C15, have been identified (using the pharmacophore-mapping approach) that act as protein-protein interaction inhibitors [56]. A pharmacophore query has been generated for both the primary and dimerization interface of TLR4-MD2 and has been used to screen the ZINC database [57] by VS and molecular docking. The docked compounds were analyzed in silico for absorption, distribution, metabolism, excretion, and toxicity (ADMET) properties and SAR. The next step was protein-protein inhibitor profiling of the screened-out compounds, and two potent compounds, C11 and C15, were selected [56]. MD simulations were performed on both compounds to check the stability of the docked complex, which showed that C15 is more stable than C11 [56].

Joce et al. have performed screening against TLR4 and MD2 and identified two antagonists, one for each target protein [58]. T5342126 was identified as a TLR4 antagonist, whereas T6071187 was found to be an inhibitor of the interaction interface of TLR4-MD2. Their activity was tested in vitro in RAW cells and it was observed that both compounds—T5342126 (at 2 µM) and T6071187 (at 200 nM)—inhibit LPS-induced TLR4 signaling [58]. Recently, in another study, derivatives of T5342126 and carvedilol [59] were tested for TLR4 inhibition [60]. These compounds were subjected to SAR analyses, and thus, novel derivatives of these two compounds were designed and synthesized. By in vitro assays, one of the derivatives, 8a, was identified as the most potent inhibitor [60] and chosen for further studies. This compound significantly inhibits inflammatory cytokines (nitric oxide and TNF-α) and downregulates TLR4 expression. Molecular docking was performed to investigate the binding mode of 8a, thereby revealing that the benzyl ring of this compound binds with the hydrophobic pocket of TLR4, and the carbazole group occupies the MD2 pocket [60].

VS has been performed on a library of 130,000 compounds obtained from the ZINC database [57], and ZINC02157367 was selected as a hit according to binding affinity. The structure of this lead molecule was optimized next to design a novel and effective compound. Considering Lipinski’s rule of five and hydrophobic active-site residues of TLR4, 12 compounds were designed [61]. Flexible docking was performed to rank the ligands by their binding energies followed by MD simulations using the all-atom optimized potentials for liquid simulations (OPLSAA) force field in Gromacs to evaluate the binding mode. The MD simulations indicated the stability of the receptor-ligand complex, and compounds 24, 14, and 17 showed the highest binding energies. To confirm these in silico results, all 12 compounds were evaluated in experimental studies, and the binding affinity of the most effective compound (17) was confirmed by surface plasmon resonance analysis [61].

In another study, five lipid A analogs (FP13-17) were designed rationally, and their binding modes were analyzed against TLR4-MD2 through molecular docking [62]. The docking analyses revealed that all the compounds fit into the MD2 pocket in an antagonist manner. To confirm the results, the docked complexes were subjected to MD simulations, and the findings indicated that all the compounds were antagonists. Later, experimental analyses were conducted to check the binding and biological activity of these compounds, and the results corroborated the antagonistic behavior of all five compounds [62].

#### 4.2.2. TLR4-Inhibitory Peptides: Lead Identification & Preclinical Studies

In a recent study [63], computational techniques were utilized to enhance the stability and activity of a TLR4-inhibitory peptide identified through phage display. The PIP2 peptide inhibits TLR4-induced proinflammatory cytokines. PIP2 was cyclized via introduction of a β-lactam bridge using in silico tools; this change improved the half-maximal inhibitory concentration (IC_50_) threefold. The optimized peptide was next tested in vitro and in vivo and caused a significant reduction of inflammation in a rat model of rheumatoid arthritis. MD simulations were performed to confirm the stability of both peptides and to understand the binding mechanism [63].

Using an ab initio computational approach, novel cyclic and linear peptides for TLR4 coreceptors CD14 and MD2 have been designed [64]. These peptides were designed based on 3D structures of the target protein in ab initio design software. Schrodinger and the PyMOL molecular graphic system [65] were used for structural visualization comparison. The missing loops were modeled by the homology modeling technique. Next, cyclic and linear peptides were designed with in silico methods and synthesized in the laboratory [64]. Experimental results indicated that the cyclic peptides are rigid as compared to the linear versions. Peptides with activity values in the micromolar range were able to trigger the TLR4-mediated signaling pathway and increase IL-1β production. These agonistic peptides are patented under patent number WO2017141248 [64].

#### 4.2.3. Miscellaneous TLR4-Related Agents

Neoseptins, a new class of TLR4 agonists, have been discovered by Morin et al. [66]. Among 90,000 compounds, neoseptin-1 was identified, and SAR analyses were performed to improve its design, thus leading to the neoseptin-3 peptidomimetic. On the other hand, the activity of this peptidomimetic is species specific because it failed to show any effect on human TLR4; that is why this compound did not proceed to clinical development [66].

Aptamers are single-stranded DNA or RNA molecules having high specificity, binding affinity, and the best characteristics of antibodies and small molecules [67]. Two TLR4-blocking DNA aptamers, ApTLR#1R and ApTLR#4F, were reported recently [68]. These aptamers were picked form a random oligonucleotide pool and optimized to enhance the affinity. ApTLR#4F and its truncated form, ApTLR#4FT, were found to be highly effective and exerted long-lasting effects in patients with a stroke [68]. Aptamers are reported to be less toxic as compared to most of small-molecule drugs, thus holding promise for clinical use.

In another study, it was demonstrated that heparin-based nanoparticles bind to TLR4-MD2 and inhibit NF-ĸB activation in macrophages [69]. With the help of computational techniques, the binding mode of the nanoparticle and TLR4-MD2 complex was analyzed, and the interacting residues of TLR4 and MD2 were highlighted [69].

A machine-learning Cox proportional hazards regression model has been employed to analyze the influence of disease marker genes on the survival of patients with ovarian cancer. In this regression analysis involving 41 genes, the expression of five genes (*TLR4*, *BSCL2*, *CDH1*, *ERBB2*, and *SCGB2A1*) accounted for significant variation in the patients’ survival rate. This study suggests that *TLR4* along with the other four genes is a potential drug target in ovarian cancer and that their expression is related to patient survival [70].

### 4.3. MD Simulations of TLR4

MD simulations were performed on the TLR4-MD2 complex with a bound natural ligand, ursolic acid (URA), which interferes the LPS binding [71]. URA is a lipophilic five ringed structure and plant-based natural compound. This study uncovered the important residues (Ile52, Leu54, Leu78, Ile80, Val82, Phe119, Phe121, Tyr131, and Cys133) for the interaction of URA and TLR4-MD2 on the basis of binding energy calculations and energy decomposition analysis. The binding mode of the inhibitor with the TLR4-MD2 complex was studied too. The diameter of the TLR4-MD2-URA complex after 150 ns MD simulations was estimated. The average diameter of the TLR4-MD2 complex was 4.43 nm, whereas in the presence of URA, the diameter diminished to an average value of 3.46 nm [71].

Recently, MD simulations were carried out to gain insight into the activation mechanism of TLR4-MD2 mouse protein structure. A 1.2 µs simulation was performed four times on four different complexes (TLR4-MD2 heterodimer, TLR4-MD2 homo-heterodimer, LPS-TLR4-MD2 homo-heterodimer, and neoseptin-3-TLR4-MD2 homo-heterodimer) to verify the stability of the complexes along with binding energy calculations [72]. The results showed stable interfaces and well-maintained structure of TLR4. Using molecular mechanics, Poisson–Boltzmann surface area (MM-PBSA) key residues were identified that play a crucial part in the dimerization and intracellular signaling of TLR4 [72].

*Rhodobacter sphaeroides* lipid A (RsLA)-induced TLR4-MD2 signaling has been studied by computational methods in different species (humans, horses, murine, and hamsters) [73]. MD simulations revealed that the RsLA backbone acquired an antagonist-like orientation in the murine and human TLR4-MD2 complex and inhibits downstream signaling. By contrast, it activates the TLR4 pathway by acquiring an agonist-like conformation in the hamster and horse complexes [73]. This dual behavior of LA is due to the binding orientation. During simulations, acyl chains in horse and hamster complexes folded back due to increased shift in the molecule. In addition, the spatial arrangement of G1cN1-G1cN2 in RsLA resembles lipid IVa in murine and human complexes. This structural change is responsible for the specific ligand behavior. Moreover, the stability of the Phe126 loop in MD2 was assessed, which is crucial for the activation of the TLR-MD2 complex. It was noted that this loop is stable in hamsters and horses as compared to murine and humans. These results provide a convincing explanation for the species-specific behavior of RsLA [73]. The importance of the Phe126 loop was reported in another study too [74]. It was demonstrated there that morphine and morphine-3-glucuronide each interacts with MD2 near this loop, thereby forming a complex, and its stability increases when it interacts with TLR4.

Recently, an interaction between HMGB1 and the TLR4-MD2 complex was studied by molecular docking and MD simulations [75]. In this study, crystal structure (PDB ID: 3FXI) of the TLR4-ECD was used. HMGB1 consist of 215 residues divided into two DNA-binding domains termed as A-box and B-box and a C-terminal domain. The cysteine residues present in the DNA-binding domains makes a disulfide bridge and induces structural changes [76]. Protein-protein docking of HMGB1, MD2, and TLR4 was performed on docking server ZDOCK [77]. MD simulations were conducted to characterize the behavior of full-length HMGB1, docked complexes of TLR4, and mutants by means of the OPLS force field. Mutagenesis and surface plasmon resonance analyses were conducted to study the interactions. Their data revealed that the N terminus of TLR4 binds to HMGB1 A-box but does not help with dimer formation, thereby preventing the launch of downstream signaling and HMGB1-induced inflammation. Meanwhile, the B-box fragment of HMGB1 promotes the TLR4 dimerization, which results in the activation of the downstream proinflammatory signaling cascade and cytokine production [75].

## 5. The Molecular Understanding of TLR4-targeting Drugs

Given the role of TLR4 in the pathogenesis of many diseases, several therapeutic modulators have been devised to regulate TLR4 expression, and a few of these compounds are currently being evaluated in clinical trials (Table 2). These compounds can be categorized as antibodies, small-molecule inhibitors, peptides, microRNAs, nanoparticles, lipid A analogs, and derivatives of natural products. Detailed information about TLR4 modulators can be found in the literature [1,60,78,79,80]. Most TLR4 agonists belong to the class of glycolipids that are LPS mimetic. LPS contains lipid A, which is a membrane-anchoring and biologically active moiety. It is composed of six fatty acid chains, two negatively charged phosphate esters, and a glucosamine disaccharide core [81]. Five of the six fatty acid chains are completely submerged into the hydrophobic pocket of the MD2 protein. Meanwhile, the other chain participates in a hydrophobic interaction with the other domains of the TLR4 molecule.

**Figure 5:**
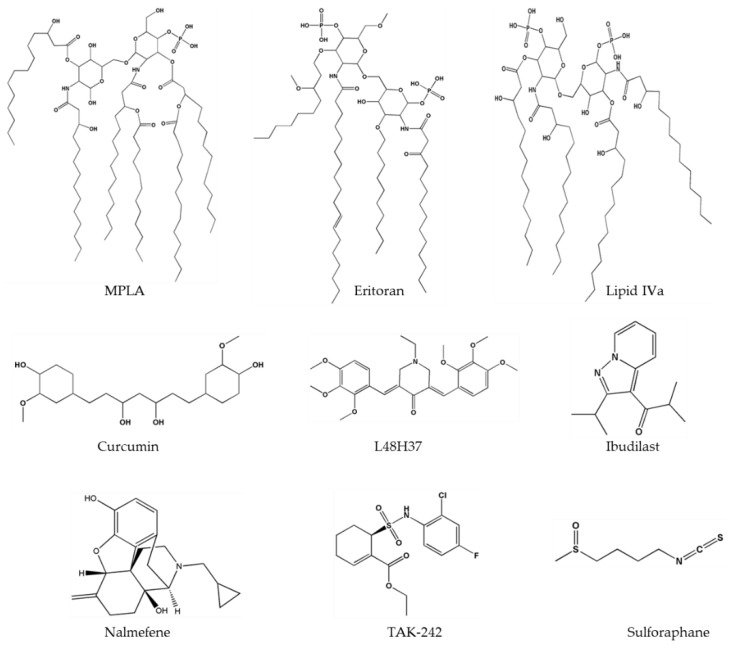
Two-dimensional structures of important TLR4 ligands.

Based on the binding mode of LPS to the TLR4-MD2 heterodimer, several strategies have been implemented to chemically modify lipid A structure, e.g., the removal of phosphate groups or a change in the position, number, or length of carbon chains or in the glucosamine backbone. These modifications can switch the activity of lipid A from an agonist to antagonist mode. The compounds derived from lipid A structure are eritoran [82], aminoglycosides [83], MPLA [84], and lipid A mimetics (Figure 5). Eritoran is a well-known TLR4 antagonist that directly binds to the hydrophobic pocket of MD2 and blocks TLR4 dimerization [13]. Nevertheless, eritoran failed in a phase III clinical trial because it yielded poor outcomes in severe sepsis cases [85]. MPLA is a TLR4 agonist (extracted from *Salmonella* Minnesota), binds to TLR4-MD2, and has been used as a vaccine adjuvant against cervical cancer and hepatitis B [86]. Other well-known lipid A analogs include lipid IVa [87] and glucopyranosyl lipid adjuvant (GLA) [88]. Lipid IVa has a species-specific activity because it acts as an agonist in mice, whereas in humans, it behaves as an inhibitor [87]. GLA is a synthetic TLR4 agonist that is composed of a phosphate group, six acyl chains, and a disaccharide backbone. It is an effective adjuvant and enhances an immune response [89,90].

The inhibition of TLR4 by a small-molecule can be achieved by blocking (1) ligand-receptor interaction, (2) dimerization of the TLR4-MD2 complex, or (3) downstream signaling. Small-molecule antagonist TAK-242 (resatorvid) (Figure 5), a cyclohexane derivative binds to Cys747 in the intracellular domain of TLR4 and inhibits its interaction with adaptor molecules [91]. A cyclohexane ring present in TAK-242 interacts with the −SH_2_ group of cysteine of TIR domain. Nonetheless, this compound failed in phase III clinical trials because it did not lower the IL-6 level in sepsis or respiratory failure [92]. Various studies have shown that curcumin has anti-inflammatory properties and downregulates pro-inflammatory cytokines such as IL-1, -2, -6, -8, and TNF-α as well as MAPKs [93]. Curcumin (Figure 5) can inhibit both pathways of TLR4 i.e., MyD88 dependent and TRIF dependent [94]—and can fit into the MD2 pocket as confirmed by experimental and computational studies [95,96,97]. To enhance the stability of curcumin, its analogs have been synthesized, and among these analogs, L48H37 (Figure 5) features greater chemical stability and enhanced anti-inflammatory properties [98]. These compounds contain a conserved group 3-(4-hydroxyphenyl) acrylaldehyde, which directly binds to the MD2 pocket and blocks the LPS binding. Therapeutic applications of curcumin in a wide range of inflammatory diseases have been reported [99]; the same is true for metabolic syndrome [100], type II diabetes mellitus [101], systemic lupus erythematosus [102], melanoma [103], pulmonary diseases, and lung cancer [104]. Another well-known plant-derived inhibitor of TLR4 is sulforaphane (Figure 5), which inhibits TLR4 dimerization and has an anti-inflammatory effect [105]. It has been reported that sulforaphane inhibits LPS-induced secretion of cyclooxygenase 2, TNF-α, HMGB1, and inducible NO synthase in macrophages [106,107]. Sulforaphane binds to the cysteine residue located in the MD2 pocket at position 133, which performs an important function in the MD2-LPS interaction. Sulforaphane occupies the binding pocket and as a result disrupts the binding of LPS to the TLR4-MD2 complex [108].

In the last decade, developments in the field of therapeutic monoclonal antibodies were incredible (in terms of the number and impact) due to their high specificity and ability to treat a variety of medical conditions. NI-0101 is a humanized monoclonal antibody that interferes with TLR4 dimerization and eventually blocks its activation. NI-0101 has strong anti-inflammatory properties and suppresses cytokine production [109]. Currently, NI-0101 is being tested in phase II clinical trials against rheumatoid arthritis (Table 2).

## 6. Conclusions

TLR4 plays a key role in the host defense mechanism and is capable of recognizing a variety of PAMPs and DAMPs. On the other hand, any dysregulation of the TLR4 pathway can initiate various diseases. Many TLR4-targeting drugs are being assessed in clinical trials, and a few of them have failed in phase III. CADD has emerged as a robust alternative approach to identify novel and potent TLR4 modulators. Computational techniques are cost effective, efficient and can be used to optimize a lead compound. Further, computational techniques such as protein modeling, protein-protein docking, and MD simulations have greatly contributed to the understanding of poorly studied crucial interfaces that may be helpful for drug discovery. Recent advances in the pharmaceutical industry are facilitating the development of safe and potent drugs.

## Figures and Tables

**Table 1:** 3D structures of human TLR4 domains available in PDB.

PDB ID	Method	Resolution	Length	Domain	Ligand	Mutations	Ref.
2Z62	X-ray	1.70 Å	27–228	TLR4-ECD/MD2	Eritoran	-	[13]
2Z63	X-ray	2.00 Å	27–527	TLR4-ECD/MD2	Eritoran	-	[13]
2Z65	X-ray	2.70 Å	27–228	TLR4-ECD/MD2	Eritoran	-	[13]
2Z66	X-ray	1.90 Å	381–627	TLR4-ECD/MD2	Eritoran	-	[13]
3FXI	X-ray	3.10 Å	27–631	TLR4-ECD/MD2	LPS	-	[12]
3UL7	X-ray	2.37 Å	28–226	TLR4-ECD	-	F63W	[50]
3UL8	X-ray	2.50 Å	27–228	ECD	-	V134L	[50]
3UL9	X-ray	2.45 Å	28–228	ECD	-	M141E	[50]
3ULA	X-ray	3.60 Å	27–228	ECD	-	F63W	[50]
4G8A	X-ray	2.40 Å	23–629	TLR4-ECD/MD2	LPS	D299G/T399I	[51]
5NAM	NMR	-	623–670	TM Domain	-	-	[17]
5NAO	NMR	-	623–657	TM Domain	-	-	[17]

**Table 2:** TLR4-targeting ligands currently in clinical trials.

Compound	Class	Disease Model	Activity	Phase	Identifier/Status
Eritoran	Glycolipid	Insulin sensitivity	Antagonist	II	NCT02321111/Completed ^3^
GLA-SE ^1^	Glycolipid	Stage III & IV soft tissue sarcoma	Agonist	I	NCT02180698/Completed
GLA (Mart-1 Antigen)	Glycolipid	Stage IIA, IIB, IIC, IIIA, IIIB, IIIC & IV melanoma	Agonist	I	NCT02320305/Active
GLA-SE	Glycolipid	Metastatic colorectal cancer	Agonist	I	NCT03982121/Active
GSK1795091	Glycolipid	Cancer, neoplasms	agonist	I	NCT02798978/Completed
NI-0101	Antibody	Rheumatoid arthritis	antagonist	II	NCT03241108/Completed
G100	Glycolipid	Follicular low-grade non-Hodgkin’s lymphoma	Agonist	I	NCT02501473/Active
CX-01	Polysaccharide	Myelodysplastic syndrome, AML ^2^	Antagonist	I	NCT02995655/Completed
GLA-SE	Glycolipid	Merkel cell carcinoma	Agonist	I	NCT02035657/Completed
G100	Glycolipid	Lymphoma	Agonist	II	NCT03742804/Not yet recruiting
GLA-SE	Glycolipid	Schistosomiasis	Agonist	II	NCT03041766/Completed
GLA	Glycolipid	Hookworm infection	Agonist	I	NCT01717950/Completed
JKB-121	Small molecule	Nonalcoholic steatohepatitis	Antagonist	II	NCT02442687/Completed
Ibudilast	Small molecule	Methamphetamine use disorder	Antagonist	II	NCT01860807/Completed

^1^ GLA: glucopyranosyl lipid A; ^2^ AML: acute myeloid leukemia; ^3^ NCT: national clinical trial.
